# Application of human lymphoid cells for the evaluation of antivirals against human adenovirus type 19: Zalcitabine has superior activity compared to cidofovir

**DOI:** 10.1177/2040206620921319

**Published:** 2020-04-28

**Authors:** Kohsuke Nakagawara, Hironori Hayashi, Kumi Kawaji, Mina Sasano, Eiichi N Kodama

**Affiliations:** 1 Division of Infectious Diseases, International Institute of Disaster Science, Graduate School of Medicine, Tohoku Medical Megabank Organization, Tohoku University, Miyagi, Japan; 2 Biochemicals Division, Yamasa Corporation, Choshi, Japan; 3 Department of Intelligent Network for Infection Control, Graduate School of Medicine, Tohoku University, Miyagi, Japan

**Keywords:** Adenovirus, screening, antiviral, pandemic, disaster

## Abstract

Human adenovirus type 19 (HAdV-19) is a major cause of the epidemic keratoconjunctivitis. Outbreaks of keratoconjunctivitis are problematic to human health, especially for infants, the elderly, and immunocompromised individuals. However, the development of anti-HAdV drugs has been hampered by inconvenient screening systems; therefore, development of a simple screening method is highly desirable. In this study, we identified that HAdV-19 can infect a human lymphoid cell line transformed with human T-cell leukemia virus (MT-2 cells). MT-2 cells supported HAdV-19 replication and showed apparent cytopathic effects within five days post-infection. Using a thiazolyl blue tetrazolium bromide (MTT)-based colorimetric assay on MT-2 cells, we were able to detect the anti-HAdV-19 activities of previously reported nucleoside/tide compounds, including (S)-1–(3-hydroxy-2-phosphonylmethoxypropyl)cytosine (cidofovir), 2′,3′-dideoxycytidine (zalcitabine) and 3′-deoxy-3′-fluorothymidine (trifluridine). Compared with previous methods, this system represents a more simple and rapid method to screen anti-HAdV-19 agents.

## Introduction

Human adenoviruses (HAdV) cause various mucosal infections, including respiratory infections, gastroenteritis, and hemorrhagic cystitis.^1^ While most infections are acute and cause severe symptoms, most patients have a good prognosis. However, HAdV infections can induce severe and lethal disseminated diseases in immunocompromised individuals. Additionally, ocular HAdV infection causes epidemic keratoconjunctivitis (EKC). HAdV type 19 (HAdV-19) is a major etiological agent of EKC, a severe and contagious infection associated with blurred vision and irritation. EKC outbreaks are problematic when they emerge in hospitals, schools, and other communities. A number of compounds, such as cidofovir (CDV), zalcitabine (ddC), and ribavirin (RBV), reportedly show anti-HAdV activity in vitro or in vivo.^2^ A newly developed compound, brincidofovir, a promising lipid-linked derivative of CDV, has been used in clinical trials.^3^ However, no anti-HAdV agents have been clinically approved.

The anti-HAdV activity of compounds has been examined in vitro using adherent cells, such as A549, HEp-2, and human embryonic lung (HEL) fibroblast cells.^4–6^ These antiviral assays can require considerable effort to change culture media. Additionally, the trypsin treatments or digestions, cell loss, and other procedures can create variation in assay results. In this study, we developed an anti-adenoviral assay using non-adherent cells to avoid these problems. We demonstrated that MT-2 cells, which are a human lymphocytic cell line transformed with human T-cell leukemia virus (HTLV-1), support the infection and propagation of HAdV-19. Cytopathic effects (CPE) of HAdV-19 infection on MT-2 cells were also evaluated by testing the infected cells for viability using a thiazolyl blue tetrazolium bromide (MTT) assay. We tested several classes of nucleoside/tide analogues such as CDV, ddC, RBV, ganciclovir (GCV), and 3′-deoxy-3′-fluorothymidine (FdT) that are known to inhibit HAdV.^2,6–10^ Except FdT, our MTT assay results agreed with previous reports on the anti-HAdV activities of these compounds. These results suggest that screening HAdV inhibitors using an MTT assay on MT-2 cells may be a useful tool for the development of novel HAdV inhibitors.

## Materials and methods

### Cell cultures and virus

A human lymphoid cell line, MT-2 (a kind gift from Dr. Shiro Shigeta, Fukushima Medical University, Fukushima, Japan), was grown in RPMI 1640 medium (Sigma-Aldrich Japan, Tokyo, Japan) supplemented with 10% fetal calf serum (FCS; Gibco, Thermo Fisher Scientific, Tokyo, Japan), 2 mM L-glutamine, 100 units/ml of penicillin, and 100 µg/ml of streptomycin (Meiji Seika Pharma Co. Ltd, Tokyo, Japan). A549 cells (procured from the RIKEN BRC through the National BioResource Project of the MEXT/AMED, Japan) were grown in DMEM (Sigma) supplemented with 10% FCS, 2 mM L-glutamine, 100 units/ml of penicillin, and 100 µg/ml of streptomycin. Cells were incubated at 37°C in a humidified atmosphere with 5% CO_2_. HAdV-19 (ATCC, Manassas, VA) was propagated in A549 cells and stored at −80°C. Mean titers were calculated and expressed as median tissue culture infectious dose per ml (TCID_50_/ml).

### MTT-based antiviral and cytotoxic assay

To evaluate anti-HAdV activity, the RPMI 1640- or DMEM-based assay medium, for MT-2 or A549 cells, respectively, with (S)-1–(3-hydroxy-2-phosphonylmethoxypropyl) cytosine (HPMPC), ddC, FdT, RBV, and GCV (Sigma-Aldrich Japan), was added in duplicate to wells on flat-bottom 96-well plates. MT-2 cells and A549 cells were mixed with HAdV-19, 2× 10^5^ cells/mL with 10^4^ TCID_50_/mL and 1× 10^5^ cells/mL with 3 × 10^3^ TCID_50_/mL, respectively, in plate wells in the presence or absence of various concentrations of the compounds (MOI: 0.035 for MT-2 cells and 0.021 for A549). The plates were incubated for five days. At the end of the incubation period, CPE was determined using the MTT assay, as described previously.^11,12^ Thus, 25 µl of the MTT solution (7.5 mg/ml) in phosphate-buffered saline (Wako, Japan) was added to each well of the plate. Then, the plate was incubated at 37°C for 2 h. After incubation, 150 µl of medium was removed with care in order not to draw cells. To solubilize the formazan crystals and neutralize the viral infectivity, 100 μl of acidified isopropanol (4 ml concentrated HCl per 1 l of isopropanol) containing 10% (v/v) Triton X-100 was added to each well. Formazan crystals were completely solubilized by pipetting up and down, the absorbance at 560 nm of the wells was read by microplate reader (GloMax, Promega, Japan). The 50% antiviral effective concentration (EC_50_) was defined as the drug concentration that protects 50% of virus-infected cells from virus-induced cell damage and/or death.^12^ The cytotoxicity of each compound was measured in parallel using the MTT assay. The 50% cytotoxicity concentration (CC_50_) was defined as the drug concentration that reduces cell viability by 50%. Data shown represent mean EC_50_ and CC_50_values (±1 standard deviation) derived from the results of two to four independent experiments conducted in duplicate.

### Quantitative PCR for detecting viral DNA from HAdV-19

A set of primers synthesized by Echavarria et al.^13^ that amplify a 139-bp region of the hexon gene (set II) was used, as previously described. Thermal Cycler Dice Real Time System Lite (Takara Bio, Kusatsu, Japan) was used for DNA amplification.

## Results

### Cytopathic effects of HAdV-19 infection against MT-2 cells

**Figure 1.**
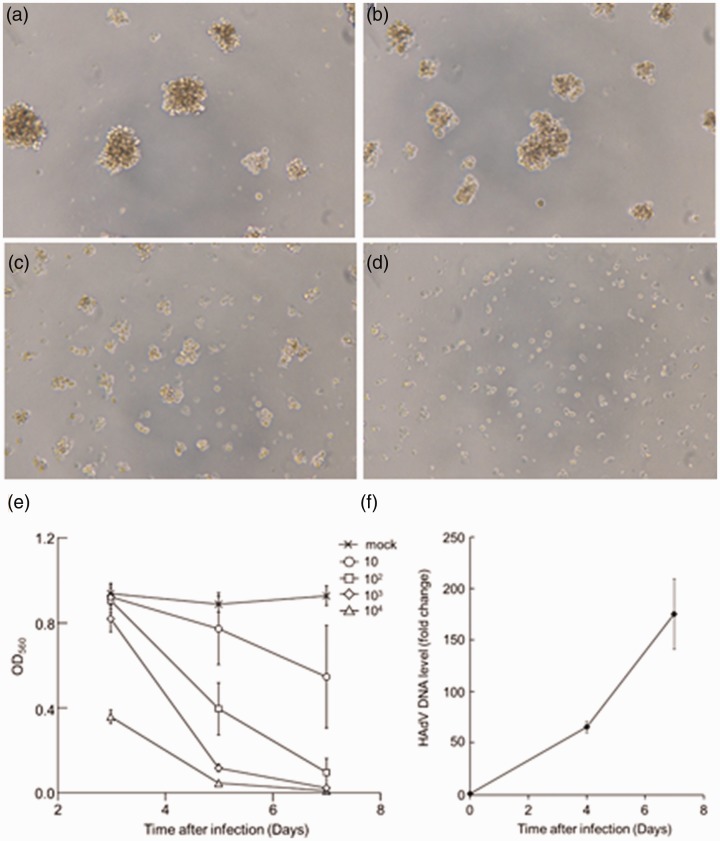
Cell viability and viral replication in HAdV-infected MT-2 cells. (a to d) MT-2 cells five days post infection with HAdV-19. Cell death was observed in a dose-dependent manner. (a) mock-infected, (b) 10^2^ TCID_50_, (c) 10^3^ TCID_50_, (d) 10^4^ TCID_50_. (e) inhibition of formazan formation in virus-infected and mock-infected MT-2 cells. Formazan formation by mock-infected, and HAdV-19-infected MT-2 cells was monitored for seven days after infection. (f) The qPCR detection of HAdV-19 in culture medium. MT-2 cells were infected with HAdV-19 for four or seven days before qPCR detection of HAdV-19 DNA in culture supernatant. HAdV DNA levels are shown as fold change for each day after infection compared with the amount at 0 days post-infection. After four and seven days of infection, the amount of viral DNA increased by more than 50 and 150 times compared with the initial amount of viral DNA, respectively.

To examine the infectivity and CPE of HAdV-19, we microscopically observed HAdV-19-infected MT-2 cells. CPE was observed in a dose-dependent manner after five days of HAdV-19 infection at 10^2^, 10^3^, and 10^4^ TCID_50_. A 100% CPE was observed at 10^3^ and 10^4^ TCID_50_ (Figure 1(c) and (d)). To determine the time course of HAdV-19-induced CPE, we examined the inhibition of formazan formation in MT-2 cells on days 3, 5, and 7 post-infection. The optical density at 560 nm (OD_560_) values decreased in a time-dependent manner (Figure 1(e)). On day 5, the OD_560_ values had decreased by approximately 80%, 40%, 20%, and 10% compared with the control, upon infection with HAdV-19 at 10^4^, 10^3^, 10^2^, and 10 TCID_50_ per well, respectively (Figure 1(e)). The Z′ factor was 0.94 when the cells were infected with 10^3^ TCID_50_ of HAdV-19, indicating robustness for screening. Therefore, we incubated 10^3^ TCID_50_ of HAdV-19 with cells for five days prior to the MTT antiviral assay.

### Detection of HAdV-19 by qPCR

To confirm HAdV-19 replication in MT-2 cells, we determined the amount of viral DNA in the culture supernatant of infected cells using qPCR methods.^4^ MT-2 cells were infected with 10^3^ TCID_50_ of HAdV-19 for four or seven days. As shown in Figure 1(f), the amount of detected viral DNA increased in a time-dependent manner. Seven days post-infection, viral DNA increased more than 150 times compared with the initial amount of viral DNA. Thus, HAdV-19 efficiently replicated in MT-2 cells.

### Effect of antiviral compounds against HAdV-19

The antiviral activities of test compounds (CDV, ddC, FdT, RBV, and GCV) were evaluated against HAdV-19 using an MTT assay with MT-2 cells (Table 1). FdT and ddC inhibited HAdV-19 replication with EC_50_ values of 1.8 and 2.6 µM, respectively. The EC_50_ values of CDV and GCV were comparable at 62 and 60 µM, respectively. In contrast, RBV showed little antiviral activity against HAdV-19. For comparison, we also evaluated the EC_50_ and CC_50_ of the compounds using A549 cells by MTT assay.^2,14^ The results showed a similar tendency. Thus, FdT and ddC exerted more potent activity than CDV and GCV. In our assay, CDV, ddC, and GCV were tolerated by MT-2 cells at a concentration of 100 µM as was the case with A549 cells. CC_50_ values of FdT and RBV against MT-2 cells (36 and 18 µM, respectively) were similar values to A549 cells (7.6 and 53 µM, respectively).

**Table 1.** Antiviral and cytotoxic effects of tested compounds against HAdV-19 determined by MTT assay.

	EC_50_ (µM)		CC_50_ (µM)		SI	
Compounds	MT-2	A549	MT-2	A549	MT-2	A549
CDV	62 ± 26	4.2 ± 1.1	>100		>1.6	>24
ddC	2.6 ± 0.4	0.35 ± 0.04	>100		>38	>286
FdT	1.8 ± 0.2	0.03 ± 0.01	36 ± 5	7.6 ± 4.2	20	>253
RBV	>18	>53	18 ± 1	53 ± 21	NA	
GCV	60 ± 7	54 ± 27	>100		>1.7	>1.9

Note: All assays to determine EC_50_ were conducted two to four times independently. The results are shown as means ± 1 standard deviation. The selectivity index (SI) is the ratio of CC_50_ and EC_50_ values.

CDV: cidofivir; ddC: zalcitabine; FdT: 3′-deoxy-3′-fluorothymidine; RBV: ribavirin; GCV: ganciclovir.

## Discussion

Most HAdV infections are severe but self-limited, even without a specific therapy. However, HAdV infection in immunosuppressed patients can cause life-threating illnesses. Additionally, ocular HAdV infection is important because it can cause EKC. Despite this, there are no approved anti-HAdV agents.

The development of anti-HAdV agents has been hampered by problematic antiviral assay methods. Plaque assays have been conventionally used for detecting HAdV infectivity and the anti-HAdV activity of compounds.^13,15,16^ Additionally, qPCR methods for detecting HAdV were developed and have been useful in analyzing HAdV infection, spread or replication, and time dependence.^4^ However, plaque assays and qPCR methods are not convenient for large-scale screening of antiviral agents. In contrast, the development of anti-HIV-1 and anticancer drugs has proven that MTT assays are well suited for drug screening. To date, several MTT methods for screening anti-HAdV agents have been developed.^12,17–20^ However, these assays use adherent cells such as A549, HEp-2, and HEL fibroblast cells, and thus require more than one week to complete and significant labor to change culture medium.^21^ In this report, we have demonstrated that HAdV-19, which was a representative pathogen causing EKC, could infect the human lymphoid cell line, MT-2, although the relationships between lymphoid cells and HAdV-19 pathogenesis have not been clarified, yet. Additionally, we established an anti-HAdV screening method with MT-2 cells using an MTT assay. MT-2 cells could be completed in five days without medium exchange. The anti-HAdV activities of compounds measured by the MTT assay on MT-2 cells showed a similar tendency to those on A549 cells. However, it is well known that the anti-HAdV activities of several compounds are serotype- and cell line dependent.^2,3,8,11^ Indeed, our method detected an EC_50_ value of FdT (1.8 µM) that is 10 to 100 times higher than the previously reported EC_50_ values determined in HAdV-2 and -3-infected Fogh and Lund (FL) cells,^22^ which were originally obtained from normal amnion but were subsequently believed to be HeLa cell contamination. Thus, the discrepancies between the reported EC_50_ values for FdT might result from the different HAdV serotypes and/or cell lines used in each study. These findings suggest that an MTT assay with MT-2 cells could be a useful tool for high-throughput screening of anti-HAdV agents. This simple, rapid, and convenient method can accelerate and enhance the development of anti-HAdV agents.
